# Interpersonal Neural Synchronization During Cooperative Behavior of Basketball Players: A fNIRS-Based Hyperscanning Study

**DOI:** 10.3389/fnhum.2020.00169

**Published:** 2020-06-24

**Authors:** Lin Li, Huiling Wang, Huiyu Luo, Xiaoyou Zhang, Ruqian Zhang, Xianchun Li

**Affiliations:** ^1^Key Laboratory of Adolescent Health Assessment and Exercise Intervention of Ministry of Education, East China Normal University, Shanghai, China; ^2^College of Physical Education and Health, East China Normal University, Shanghai, China; ^3^Shanghai Yucai Junior High School, Shanghai, China; ^4^School of Psychology and Cognitive Science, Shanghai Changning-ECNU Mental Health Center, East China Normal University, Shanghai, China

**Keywords:** cooperation, basketball, hyperscanning, brain synchronization, fNIRS

## Abstract

Accumulating evidence has consistently shown that team-based sports (such as basketball) are beneficial to interpersonal cooperation. However, its neural correlate remains to be discovered, especially in the perspective of two-person neuroscience. In this study, 12 dyads of basketball players and 12 dyads of college students who had no experience of team-based sports training were asked to perform joint-drawing task and control task. During task performance, neural activities were recorded in frontal area by the functional near-infrared spectroscopy (fNIRS)-based hyperscanning approach. The results demonstrated that dyads of basketball players were faster to finish joint-drawing task and showed higher subjective cooperativeness than dyads of college students. Meanwhile, significant interpersonal neural synchronization (INS) was observed in the dorsolateral prefrontal area only when pairs of basketball players performed joint-drawing task, but not control task. Therefore, we provide the first piece of inter-brain evidence for enhanced cooperative behavior in the individuals with team-based sports training, which could make us deeply understand exact neural correlate for experience-dependent changes of cognitions in humans.

## Introduction

Cooperation is a joint action between individuals or groups that enables achievement of common goals while people collaborate with each other (Fantasia et al., 2014). There are two main types of cooperation: one is joint action and the other is joint decision-making. The researcher proposes that joint action refers to the coordination of individuals’ respective behaviors in the time and space dimensions in order to achieve a common goal (Sebanz et al., 2006), such as synchronized dance, synchronized singing, and imitation. While joint decision-making refers to two or more people making decisions separately in the same situation, and their respective decisions will affect the results of themselves and their partners (Hasson et al., 2012). A wide range of sports contain cooperative behaviors, such as basketball, table tennis doubles, and so on, in which two or more people cooperatively work together to achieve common goals. Thus, performance in those sports should be linked with the level of interpersonal cooperation during the game.

Basketball is a highly competitive team sport between two teams that requires multiple people to participate in. The main characteristic of basketball is that the team’s goal is achieved by the tacit interaction and cooperation between athletes in a highly tense and rapidly changing environment (Li, 2015). In this game, the players of the offensive team pass the ball, cooperate with one another to cover each other, and break through the opponent’s defense. The defense also needs coordination through zone or man-to-man defense to block the offense from scoring. No matter joint action or joint decision-making in offensive and defensive processes, both of them can be regarded as interpersonal cooperation. The characteristics of basketball suggest that it can improve people’s cooperation and prosocial behaviors. A questionnaire study assessed 16 personality factors of 103 Chinese male (Sheng, 1999) and 77 female basketball players (Hou and Sheng, 2001). They found that basketball players were more passionate and courageous, as well as more cooperative and adaptable. Another questionnaire study found that compared with students who did not play basketball regularly, those who often played it were more obedient and got along better with others. Furthermore, basketball could promote handicapped people’s mental health and social skills (Fiorilli et al., 2013). All these findings consistently indicate the positive effects of basketball on cooperative behaviors in the perspective of personality. However, hitherto, the neural mechanism underlying basketball players’ cooperation still remains to be clarified.

Hyperscanning refers to simultaneous neural recording from two or more socially interacting individuals (Montague et al., 2002). It can provide inter-brain evidence for cognitions enriched with interactions. The interpersonal neural synchronization (INS) has been proven to be an important neural marker for various social interactions, such as joint action (Bilek et al., 2015, 2017; Osaka et al., 2015), teaching (Holper et al., 2013; Dikker et al., 2017; Zheng et al., 2018; Pan et al., 2020; Sun et al., 2020), imitation (Dumas et al., 2010; Holper et al., 2012; Pan et al., 2018), and communication (Stephens et al., 2010; Jiang et al., 2012; Dai et al., 2018). Many types of paradigms have been employed in exploring cooperation in the hyperscanning studies, including key-pressing task (Cui et al., 2012; Pan et al., 2017), collaborative mapping (Cheng et al., 2019), and collaborative decision making tasks such as prisoner’s dilemma (Hu et al., 2018) and ultimatum game (Tang et al., 2016). In these studies, the INS in prefrontal area or temporal-parietal junction is consistently observed to be much higher when dyads of subjects perform a cooperation task, not control task (Lindenberger et al., 2009; Metzger et al., 2017). Moreover, INS is positively correlated with cooperative performance (Cui et al., 2012; Pan et al., 2017).

Therefore, we attempt to explore the effect of basketball training on cooperation in the framework of two-person neuroscience. Dyads of basketball players or college students (as control) were asked to perform a joint-drawing task and control task, during which prefrontal activities were simultaneously recorded from each dyad of subjects. Based on previous findings, we hypothesized that basketball players should have better performance in the cooperative task, which should be associated with higher INS in the prefrontal cortex.

## Materials and Methods

### Subjects

Twelve dyads of basketball players (experimental group) and 12 dyads of college students (control group) who had no experience of sports training were recruited in this study. They were all males, and at the age of around 20 (experimental group, 19.95 ± 1.43; control group, 19.7 ± 1.87). Participants in the experimental group were Chinese national second-level athletes, while participants in the control group had no experience of team-based sports training. All dyads were right-handed, with normal or corrected-to-normal vision. None of the participants had color blindness or any physical or mental illness.

In order to control the influence of intimacy on results, the dyads were required to get to know each other for at least three months before the experiment. All participants fully understood the tasks and signed the informed consent form before the experiment. The experiment was approved by the Ethics Committee of Human Experiments of East China Normal University. All participants received the remuneration after the experiment.

### Procedures and Tasks

**FIGURE 1:**
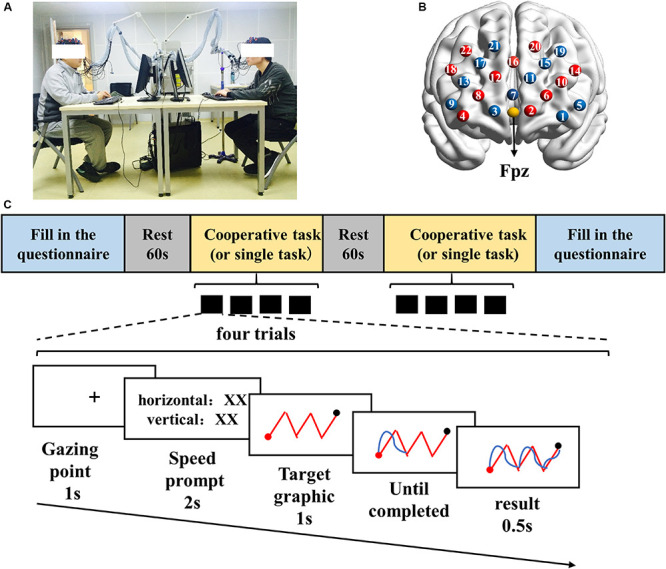
Experimental design. **(A)** Experimental scene. **(B)** Channel location. **(C)** Task procedure.

Once participants arrived in the laboratory, they filled in a questionnaire about basic information, such as age, gender, training years, and completed interpersonal intimacy scale (Aron et al., 1992) (Supplementary Figure S1). The participants were then told about the experimental procedures and tasks. Once both of them fully understood the tasks, the experiment formally began. The participants sat across the table, with two monitors in the middle (Figure 1A). After 1-min rest, the joint or single drawing task (described below) began. After finishing the tasks, participants were required to complete a task participation scale (Supplementary Figure S2) to measure the subjective cooperativeness of oneself and the partner during the task. The items were rated on a seven-point Likert-scale with one (“very low”) to seven (“very high”).

The study adopted a joint drawing task designed to measure cooperative behaviors (Arueti et al., 2013). The task required participants to control the direction (top, bottom, left, and right) of a brush on a computer screen to trace a target graphic. In the cooperative task, participant 1 controlled the vertical movement of the brush by pressing the up (“↑’) and down (“↓”) arrow keys, and participant 2 controlled the horizontal movement of the brush by pressing the left (“←”) and right (“→”) arrow keys. However, in the single task, two participants should control the up, down, left, and right directions to trace the target graphics, respectively. Participant 1 pressed the arrow keys (“↑,” “↓,” “←,” “→”), and participant 2 pressed the keys of “W,” “A,” “S,” and “D.” Each participant could only see his own movements presented on the screen in the single task.

The specific process of each trial was as follows. First, a fixation appeared in the center of the screen. Then, a prompt (i.e., “speed prompt”) was presented to indicate the moving speed of the brush in the present trial. The speed prompt could help participants adjust their key pressing speed more effectively, but it did not function as a variable in the current study. After that, the screen presented a red target graphic to the participants. In the process of drawing, the target graphic always appeared on the screen. The participants had to control the brush to move along the given path, from the starting point (the red point) to the end point (the black point). When the brush reached the end point, the screen automatically jumped to the next target graphic. With each key pressing, participants could clearly see the trajectory of the brush movement (blue curves) which helped them adjust their movement directions according to this real-time feedback (Figure 1C).

To balance the effect of order of the task, for both experimental and control groups, half of the dyads completed the cooperative task first and then performed the single task, while the other half completed the single task first and then performed the cooperative task. Each task had four trials; thus, each participant had eight trials to complete. There was no time limit to complete a trial, and the next trial did not begin until the brush moved to the end point.

### Data Collection

The joint drawing task was written and run on the MATLAB platform using Psychotoolbox (The Math Works, Inc.^1^, Psychophysics Toolbox Version 3^2^). The screen resolution of the computer monitor was 1920 × 1080 pixels, and the refresh rate was 60 Hz.

The ETG-7100 optical topography system (Hitachi Medical Corporation, Japan) was adopted to record concentration changes of oxy-hemoglobin (Hbo) and deoxy-hemoglobin (Hbr) for each dyad of participants. The 3 × 5 probe patch, inlaid in a swimming cap, was placed on each participant’s forehead, covering the prefrontal cortex. Each probe patch was composed of eight emitters and seven detectors, 3 cm apart and forming 22 channels. According to the 10-20 international system, the center detector of the middle row was placed at Fpz position (Figure 1B). The probes of the middle column were aligned to the midline, from the Nasion to the Inion. The spatial location of each channel referenced to the template provided by Jichi University^3^ (Supplementary Table S1). The wavelength of the near infrared light was 695 and 830 nm, and the sampling frequency was 10 Hz.

### Data Processing and Analysis

#### Behavioral Data Analysis

The Psychtoolbox recorded the time spent on each trial and the location of the brush in relation to the tracing line. Completion time referred to the time length required for the brush to move from the red point to the black point, and deviation area referred to the area that the brush trajectory deviated from the original target graphic (Figure 1C). The deviation area was calculated as follows: (Monitor area × the number of pixels that the trajectory deviated from the original shape)/screen resolution. The unit was cm^2^. The reciprocal of the deviation area was used to indicate the “completion effect” of the drawing task. The greater reciprocal of the deviation area, the better the completion effect. What is more, another indicator was also established to measure the quality of the performance, namely, completion efficiency, referring to the completion effect per unit time (Completion efficiency = Completion effect/Completion time) (Cheng et al., 2019).

Through SPSS Statistics 19, we conducted independent sample *t*-tests on the intimacy and task participation, and conducted 2 (Group: experimental group, control group) × 2 (Condition: single task, cooperative task) repeated measures analysis of variance on completion time and completion efficiency. In order to more objectively and accurately reflect the level of cooperation and exclude the interference of unrelated variables, we took the single-person task as the baseline, thus subtracting completion time and completion efficiency of the single-person task from those of the cooperative task. The difference values, defined as cooperation time and cooperation efficiency, were used as criteria for evaluating the quality of cooperation. A large difference corresponded to better cooperation.

#### fNIRS Data Analysis

Functional near-infrared spectroscopy (fNIRS) data were processed by MATLAB 2014a. Given that compared with Hbr, Hbo is a better indicator of changes in the cerebral blood flow (Hoshi, 2003, 2007), only Hbo signals were analyzed in the study. In order to eliminate the global components, the principal component spatial filtering (PCA) algorithm (Zhang et al., 2016) was adopted. To obtain the INS, we performed wavelet transform coherence (WTC) analysis (Grinsted et al., 2004). To identify the task-related frequency band, we calculated the time-averaged coherence at each frequency from 0.02 to 0.2 Hz as previous studies (Nozawa et al., 2016; Pan et al., 2018, 2020; Zheng et al., 2018; Liu et al., 2019). The INS of the baseline (30 s rest) was subtracted from that of the task session. After that, a series of one-sample *t*-tests were conducted on all channels. Given that this analysis was only used to identify the task-related frequency range rather than obtaining final results, therefore, no multiple comparison correction was performed. Significantly increased INS was found in the frequency band ranging from 0.048 to 0.068 Hz (14.8–21 s, Supplementary Figure S3). Combined with visual inspection on pictures of WTC for two different tasks (Supplementary Figure S4), finally, the frequency band between 0.039 and 0.078 Hz (12.8–25.6 s) was chosen as the frequency band of interest in the current study. The average coherence in this band of each task was calculated by subtracting the average coherence in the rest session from that in the task session. After converting coherence values into z-scores, we performed one-sample *t*-tests with FDR correction (Benjamini and Hochberg, 1995) to find significantly synchronized channels in each task for two different groups separately. For channels with significant INS increase in at least one condition, we performed the 2 (Group: experimental group, control group) × 2 (Condition: single-person task, cooperative task) repeated measures ANOVA. At last, the contrasts between cooperative task and single task of the experimental and control groups were compared through independent sample *t*-test.

## Results

### Behavioral Results

**FIGURE 2:**
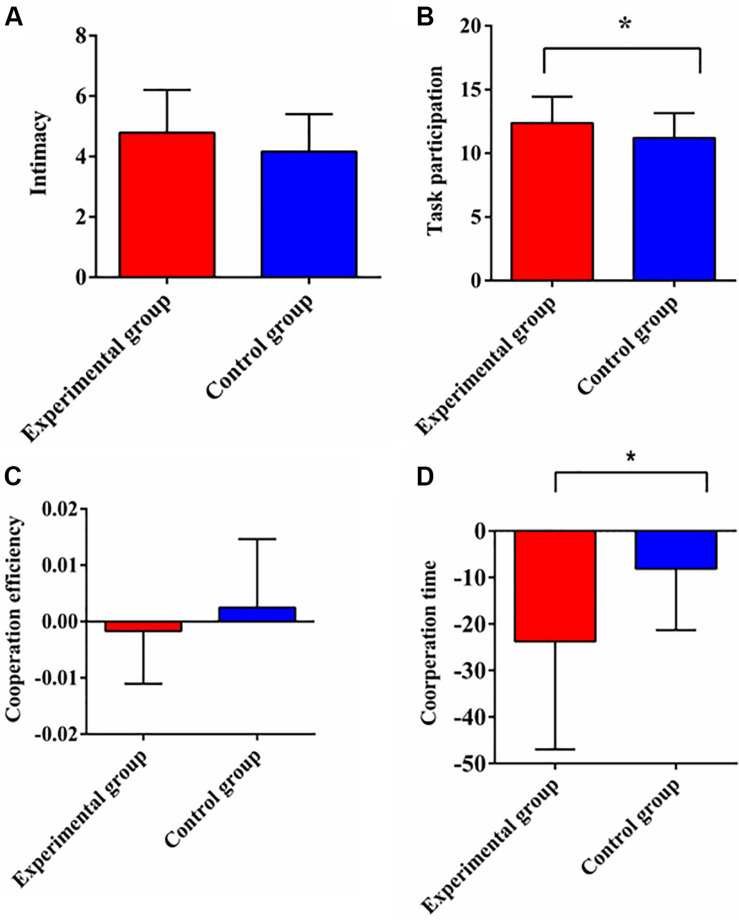
The comparisons of intimacy **(A)**, task participation **(B)**, cooperation efficiency **(C)**, and cooperation time **(D)** between experimental group and control group. Cooperation efficiency and cooperation time are the difference of completion efficiency and the difference of completion time between two tasks respectively. Note: Error bars represent standard error; *designates *p* < 0.05.

The differences of intimacy and task participation between two groups were examined by independent sample *t*-tests. The results showed that there was no significant difference of intimacy between two groups [*t*_(__46__)_ = 1.629, *p* = 0.11, Cohen’s *d* = 0.470; Figure 2A]. However, task participation in the experimental group was significantly higher than that in the control group [*t*_(__46__)_ = 2.023, *p* = 0.049, Cohen’s *d* = 0.584; Figure 2B].

The 2 (Group: experimental group, control group) × 2 (Condition: single task, cooperative task) repeated measures ANOVA on the completion time revealed significant main effects of group [*F*_(__1_,_22__)_ = 4.834, *p* = 0.039, η^2^ = 0.18] and condition [*F*_(__1_,_22__)_ = 17.675, *p* < 0.001, η^2^ = 0.446]. The interaction effect reached to the edge of a significant level [*F*_(__1_,_22__)_ = 3.966, *p* = 0.059, η^2^ = 0.153]. The simple effect analysis found that the completion time of the single task in the experimental group was longer than that of the control group, *p* = 0.034. There was no significant difference of the completion time between two groups for the cooperative task, *p* = 0.156. The analysis on completion efficiency showed that the main effect of group [*F*_(__1_,_22__)_ = 0.056, *p* = 0.815, η^2^ = 0.003], the main effect of condition [*F*_(__1_,_22__)_ = 0.03, *p* = 0.865, η^2^ = 0.001] and the interaction effect [*F*_(__1_,_22__)_ = 0.222, *p* = 0.642, η^2^ = 0.01] were not significant.

In order to examine the differences between two groups, we used the single-person task as a baseline, and performed independent sample *t*-tests on the difference of completion time and efficiency between two tasks, namely, cooperation time and cooperation efficiency. The result indicated that group had the marginal significant effect on the cooperation time [*t*_(__22__)_ = −1.991, *p* = 0.059, Cohen’s *d* = 0.813], with the cooperation time of the experimental group being shorter than that of the control group (Figure 2D). But there was no significant difference of the cooperation efficiency [*t*_(__22__)_ = −0.47, *p* = 0.642, Cohen’s *d* = 0.186; Figure 2C].

### fNIRS Results

**FIGURE 3:**
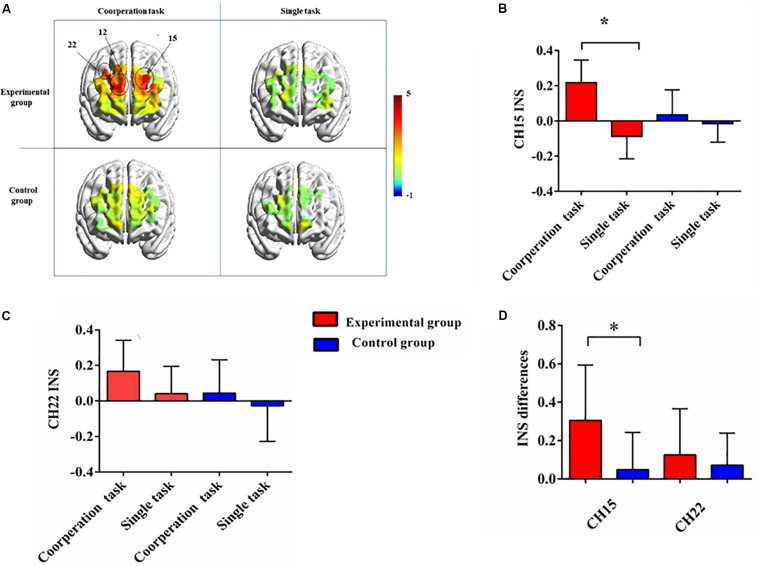
**(A)** T-maps of interpersonal neural synchronization of two groups in different conditions. For the experimental group, channel 12, channel 15, and channel 22 showed significant INS increase in the cooperative task. **(B)** The task-related INS in channel 15 of two groups in different tasks. **(C)** The task-related INS in channel 22 of two groups in different tasks. **(D)** The INS contrasts (cooperative task—single task) of two groups in channel 15 and 22. Error bars are standard errors. *designates *p* < 0.05.

We first examined the INS of the experimental group in the cooperative task. A series of one sample *t*-tests found that for channel 12 [Frontopolar, *t*_(__11__)_ = 4.021, *p* = 0.002, Cohen’s *d* = 1.161], channel 15 [Dorsolateral prefrontal cortex, *t*_(__11__)_ = 3.745, *p* = 0.003, Cohen’s *d* = 1.081], and channel 22 [Dorsolateral prefrontal cortex, *t*_(__11__)_ = 3.320, *p* = 0.007, Cohen’s *d* = 0.958], there was significant INS (Figure 3A). After FDR correction, these three channels still reached significant level (*p* < 0.05). With respect to the single task, no channel with significant INS was found. The same analysis was conducted for the control group, and no significant INS was detected in two tasks.

Then the 2 (Group: experimental group, control group) × 2 (Condition: single task, cooperative task) repeated measures ANOVA was performed for channel 12, channel 15, and channel 22. With respect to channel 12, the results showed that there were no significant main effects [group: *F*_(__1_,_22__)_ = 0.763, *p* = 0.392, η^2^ = 0.034; condition: *F*_(__1_,_22__)_ = 3.006, *p* = 0.097, η^2^ = 0.12] or interaction effect [*F*_(__1_,_22__)_ = 0.679, *p* = 0.419, η^2^ = 0.030]. For channel 15, the results showed that the main effect of group was not significant [*F*_(__1_,_22__)_ = 0.746, *p* = 0.397, η^2^ = 0.033], but the main effect of condition [*F*_(__1_,_22__)_ = 12.64, *p* = 0.002, η^2^ = 0.365] and the interaction effect were significant [*F*_(__1_,_22__)_ = 6.49, *p* = 0.018, η^2^ = 0.228]. Further simple effect analysis found that the INS of the experimental group in the cooperative task was significantly stronger than that of the control group, *p* = 0.047. However, such difference was not observed in the single task, *p* = 0.346 (Figure 3B). As for channel 22, only the main effect of condition was significant [group: *F*_(__1_,_22__)_ = 2.552, *p* = 0.124, η^2^ = 0.104; condition: *F*_(__1_,_22__)_ = 5.55, *p* = 0.028, η^2^ = 0.201; interaction: *F*_(__1_,_22__)_ = 0.406, *p* = 0.531, η^2^ = 0.018; Figure 3C].

To clear the effects of group on INS, we took the single-person task as a baseline, and performed an independent sample *t*-test on the INS difference between cooperative task and single task of channel 15 and channel 22. In channel 15, the analysis revealed a significant difference between two groups [*t*_(__22__)_ = 2.548, *p* = 0.018, Cohen’s *d* = 1.04], with the experimental group having significantly stronger INS increase than the control group. But the comparison for the INS increase in channel 22 did not showed such difference [*t*_(__22__)_ = 0.637, *p* = 0.531, Cohen’s *d* = 0.26; Figure 3D].

Furthermore, to make clear the relationship between INS and behavioral performance, Pearson correlation coefficients were calculated. However, for both groups, no statistically correlations were found between task-related INS in channel 12, 15, or 22 and completion efficiency (experimental group: *p*s > 0.10; control group: *p*s > 0.653, FDR controlled), deviation area (experimental group: *p*s > 0.507; control group: *p*s > 0.783, FDR controlled), and completion time (experimental group: *p*s > 0.782; control group: *p*s > 0.159, FDR controlled).

## Discussion

To explore the inter-brain neural mechanism of basketball players during interpersonal cooperation, the present study combined the joint drawing task and fNIRS-based hyperscanning. During the task, two participants needed to adjust their key-pressing speed and brush directions through real-time feedback presented on the screen to trace the given graphics. The behavioral results showed that although two groups performed comparatively in the cooperative task, the subjective cooperation of the experimental group was significantly higher than that of the control group. Furthermore, there was significant INS in the dorsolateral prefrontal cortex during the cooperative task for basketball players.

The paradigm used in this study was the joint drawing task. To trace the target graphics as accurately as possible, two partners needed to continuously adjust their speed of key pressing and the direction of the brush based on their partner’s performance during the task. In basketball games, for perfect cooperation, athletes not only pay attention to the situation of themselves, but also pay attention to the situation of their teammates. In this sense, interpersonal cooperation of the joint drawing task was similar to that in basketball games. Therefore, the experimental paradigm in current study was suitable for explore the difference of cooperation between basketball athletes and normal persons.

The fNIRS results showed that the basketball athletes had stronger INS in the dorsolateral prefrontal area in the cooperative task. Previous studies have shown that the dorsolateral prefrontal lobe is involved in cognitive control (Sanfey et al., 2003; Gabay et al., 2014; Feng et al., 2015), and plays an important role in cooperation-related decision-making (Fermin et al., 2016; Lukinova and Myagkov, 2016; Macoveanu et al., 2016). When dyads choose to cooperate, the dorsolateral prefrontal area is more sensitive. Using fNIRS-based hyperscanning, increased INS between two interacting persons has been observed in superior frontal cortex and dorsolateral prefrontal cortex when they performed a cooperation task (Cui et al., 2012; Pan et al., 2017). Using transcranial magnetic stimulation technology to inhibit the activity of the dorsolateral prefrontal area could reduce the cooperative behavior of the subjects (Soutschek et al., 2015). What is more, compared with cooperating with strangers, the INS of parent–child interactions was greater in the dorsolateral prefrontal area and the frontal pole cortex (FPC) in the cooperative game, which was not found in the competitive task (Reindl et al., 2018). Similar to the results of aforementioned literature, our study also found that basketball players showed significant INS in the dorsolateral prefrontal area in cooperative tasks.

In our study, we did not find significant correlation between the behavioral indicators and INS. We speculate that during the cooperation tasks, the nerves activated faster than muscles in the nerve-muscle system, and cooperation consciousness was faster than cooperative behavior. Once basketball players began to cooperate, the brain would quickly get into a cooperative state. But due to inexperience in the experimental paradigm, the cooperation level of the basketball players in behavior was not perfectly reflected. We also analyzed the task participation of the experimental group and the control group, and found that the task participation of the experimental group was significantly better than that of the control group. This also showed that the sense of cooperation of basketball players was stronger than college students who had no experience of team-based sports training.

This study also has some limitations. First, the experimental paradigm did not perfectly reflect the characteristics of basketball and the level of cooperation of basketball players was not fully displayed. Second, the fNIRS probe board was too small to cover the entire brain, leading to incomplete observation of the brain. Finally, gender is also an important factor influencing cooperation. In this study, only men were included as participants. This is an avenue for future research.

This research is the first study on cooperation in the field of sports using fNIRS-based hyperscanning technology. It attempted to observe the relationship between sports and cooperation from the perspective of cognitive neuroscience. Future research should adopt a task paradigm combined with sports scenarios, which can better reflect the ecological nature of athlete cooperation.

## Data Availability Statement

The datasets generated for this study are available on request to the corresponding author.

## Ethics Statement

The studies involving human participants were reviewed and approved by the University Committee on Human Research Protection, East China Normal University. The patients/participants provided their written informed consent to participate in this study.

## Author Contributions

LL and XL designed the research. HW and HL finished the experiment. HL, HW, and RZ analyzed the data. LL, XL, HW, and XZ wrote the draft. All authors reviewed the manuscript.

## Conflict of Interest

The authors declare that the research was conducted in the absence of any commercial or financial relationships that could be construed as a potential conflict of interest.

## Footnotes

1 https://www.mathworks.com/products/matlab.html

2 http://psychtoolbox.org/

3 http://www.jichi.ac.jp/brainlab/virtual_registration/Result3x5_E.html
