# Supplementary material for: Interpersonal Neural Synchronization During Cooperative Behavior of Basketball Players: A fNIRS-Based Hyperscanning Study
Source: Front Hum Neurosci. 2020 Jun 24;14:169. doi: 10.3389/fnhum.2020.00169 (PMC7358650; doi:10.3389/fnhum.2020.00169)
Supplement: Supplementary file 1 [file Table_1.docx]

Supplementary Material

**Supplementary Table S1.** The coordinates in MNI space and corresponding neuroanatomical labels for channels in the prefrontal cortex.

| **Channel** | **MNI coordinate** | | | **AAL** | | **Brodman Talairach** | |
| --- | --- | --- | --- | --- | --- | --- | --- |
|  | **x** | **y** | **z** | **Area** | **Percentage** | **Area** | **Percentage** |
| **1** | **-36** | **63** | **-7** | **Frontal_Mid_Orb_L** | **1.00** | **10 - Frontopolar area** | **0.86** |
| **2** | **-13** | **71** | **-3** | **Frontal_Mid_Orb_L** | **0.56** | **10 - Frontopolar area** | **0.98** |
| **3** | **15** | **71** | **-3** | **Frontal_Sup_Orb_R** | **0.83** | **10 - Frontopolar area** | **1.00** |
| **4** | **38** | **63** | **-7** | **Frontal_Mid_Orb_R** | **0.96** | **10 - Frontopolar area** | **0.84** |
| **5** | **-45** | **53** | **1** | **Frontal_Mid_L** | **0.52** | **10 - Frontopolar area** | **0.95** |
| **6** | **-24** | **68** | **8** | **Frontal_Sup_L** | **0.96** | **10 - Frontopolar area** | **1.00** |
| **7** | **2** | **68** | **9** | **Frontal_Sup_Medial_L** | **0.58** | **10 - Frontopolar area** | **1.00** |
| **8** | **27** | **68** | **8** | **Frontal_Sup_R** | **1.00** | **10 - Frontopolar area** | **1.00** |
| **9** | **47** | **53** | **2** | **Frontal_Mid_R** | **0.59** | **10 - Frontopolar area** | **1.00** |
| **10** | **-35** | **58** | **19** | **Frontal_Mid_L** | **1.00** | **10 - Frontopolar area** | **1.00** |
| **11** | **-13** | **67** | **22** | **Frontal_Sup_L** | **0.86** | **10 - Frontopolar area** | **1.00** |
| **12** | **14** | **68** | **23** | **Frontal_Sup_R** | **0.74** | **10 - Frontopolar area** | **1.00** |
| **13** | **38** | **59** | **18** | **Frontal_Mid_R** | **0.89** | **10 - Frontopolar area** | **1.00** |
| **14** | **-45** | **42** | **27** | **Frontal_Mid_L** | **0.89** | **46 - Dorsolateral prefrontal cortex** | **0.95** |
| **15** | **-23** | **56** | **33** | **Frontal_Sup_L** | **0.58** | **9 - Dorsolateral prefrontal cortex** | **0.71** |
| **16** | **2** | **59** | **34** | **Frontal_Sup_Medial_L** | **0.74** | **10 - Frontopolar area** | **0.50** |
| **17** | **26** | **57** | **33** | **Frontal_Sup_R** | **0.57** | **9 - Dorsolateral prefrontal cortex** | **0.70** |
| **18** | **47** | **42** | **28** | **Frontal_Mid_R** | **0.89** | **46 - Dorsolateral prefrontal cortex** | **0.93** |
| **19** | **-35** | **40** | **42** | **Frontal_Mid_L** | **0.92** | **9 - Dorsolateral prefrontal cortex** | **0.68** |
| **20** | **-11** | **50** | **45** | **Frontal_Sup_L** | **0.68** | **8 - Includes Frontal eye fields** | **0.73** |
| **21** | **13** | **50** | **46** | **Frontal_Sup_Medial_R** | **0.51** | **8 - Includes Frontal eye fields** | **0.70** |
| **22** | **36** | **40** | **42** | **Frontal_Mid_R** | **1.00** | **9 - Dorsolateral prefrontal cortex** | **0.60** |


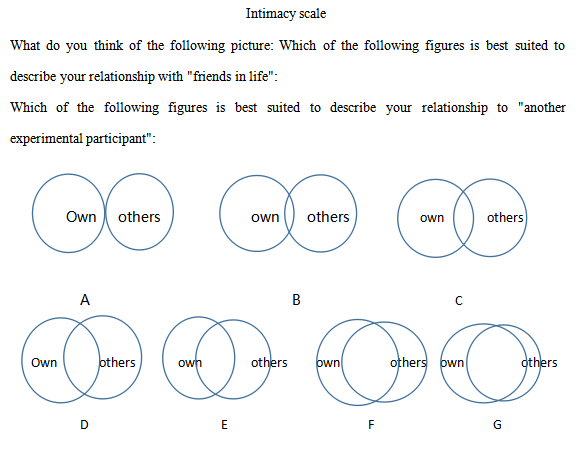


Supplementary Figure S1. The scale used to measure interpersonal intimacy. The greater overlap of two circles denotes more intimacy of relationship between two persons.


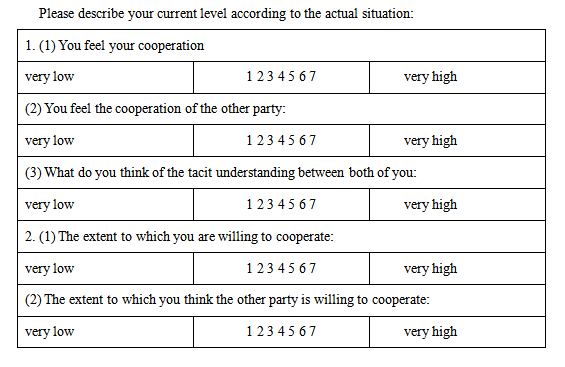


Supplementary Figure S2. The scale used to measure subjective cooperativeness of oneself and partners.


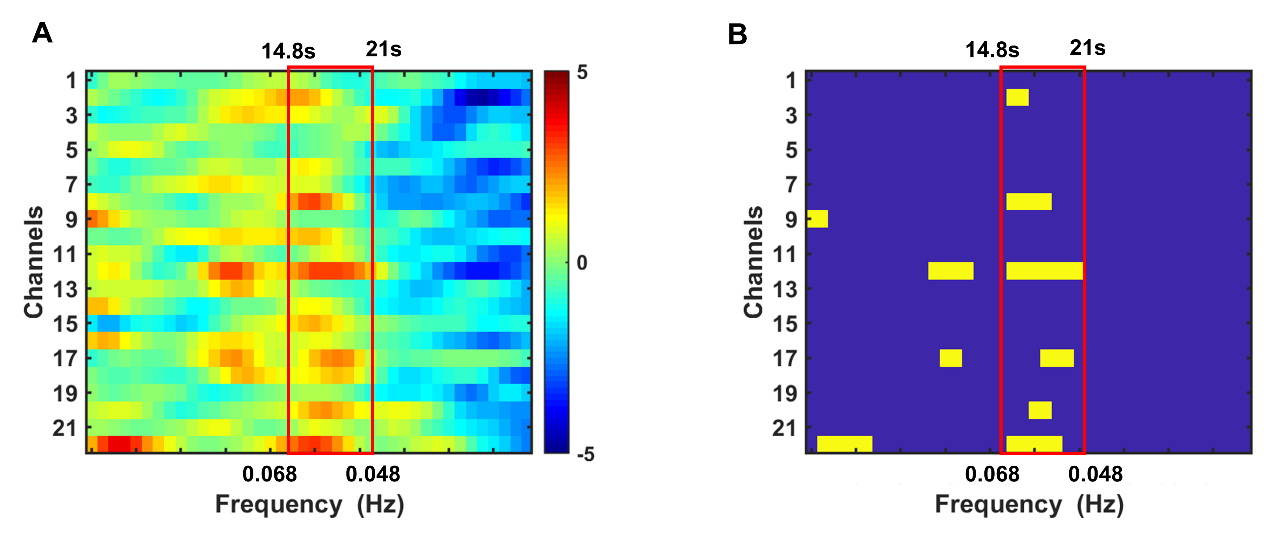


Supplementary Figure S3. (A) T-value map resulting from a series of one-sample *t*-tests conducted on task-related INS which was defined as increased coherence in the task (task – rest). (B) Channels with *p* < 0.05 are marked by yellow blocks. The red border covers the frequency band ranging from 0.048 to 0.068 Hz (14.8-21s).


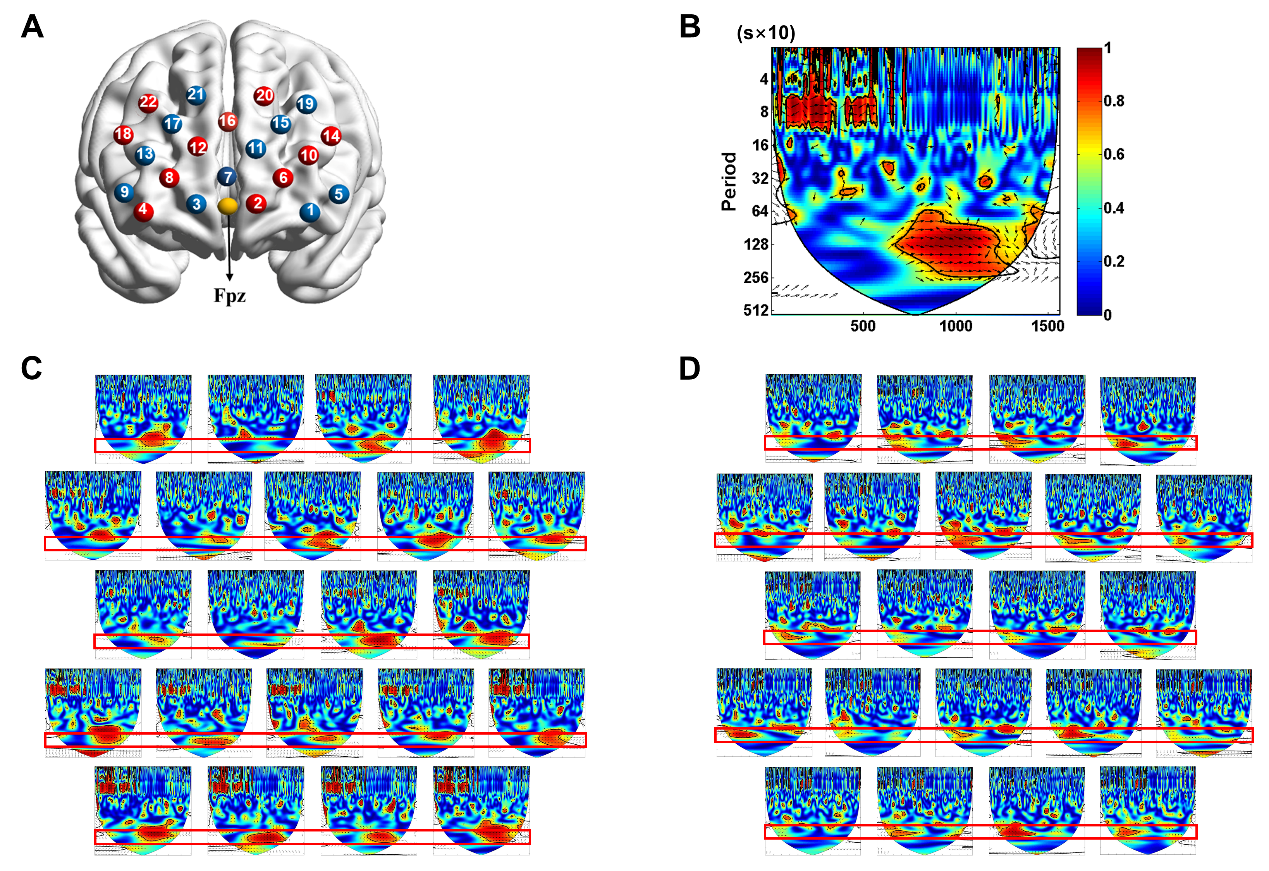


Supplementary Figure S4. (A) The location of 22 channels covering the prefrontal cortex. (B) Wavelet coherence in the first channel of a representative participant dyad. The red color denotes higher coherence. (C) Wavelet coherence of the cooperation task in all channels of a representative participant dyad. The relative position of channels is identical to that shown in Fig. B. The red borders cover the frequency band of interest (12.8-25.6s). (D) Wavelet coherence of the single task in all channels of a representative participant dyad.
